# Development of a CT Image Analysis Model for Cast Iron Products Based on Artificial Intelligence Methods

**DOI:** 10.3390/ma15228254

**Published:** 2022-11-21

**Authors:** Adam Tchórz, Krzysztof Korona, Izabela Krzak, Adam Bitka, Marzanna Książek, Krzysztof Jaśkowiec, Marcin Małysza, Mirosław Głowacki, Dorota Wilk-Kołodziejczyk

**Affiliations:** 1 Łukasiewicz Research Network–Krakow Institute of Technology, Zakopiańska 73, 30-418 Krakow, Poland; 2 Faculty of Metals Engineering and Industrial Computer Science, AGH University of Science and Technology, Al. Mickiewicza 30, 30-059 Krakow, Poland; 3 Faculty of Non-Ferrous Metals, AGH University of Science and Technology, Al. Mickiewicza 30, 30-059 Krakow, Poland; 4 Faculty of Natural Sciences, Jan Kochanowski University of Humanities and Sciences in Kielce, Ul. Żeromskiego 5, 25-369 Kielce, Poland

**Keywords:** cast iron, 3D tomography for cast metal, recommendation system, neural networks, defect analysis

## Abstract

This paper presents an assessment of the possibility of using digital image classifiers for tomographic images concerning ductile iron castings. The results of this work can help the development of an efficient system suggestion allowing for decision making regarding the qualitative assessment of the casting process parameters. Special attention should be focused on the fact that automatic classification in the case of ductile iron castings is difficult to perform. The biggest problem in this aspect is the high similarity of the void image, which may be a sign of a defect, and the nodular graphite image. Depending on the parameters, the tests on different photos may look similar. Presented in this article are test scenarios of the module analyzing two-dimensional tomographic images focused on the comprehensive assessment by convolutional neural network models, which are designed to classify the provided image. For the purposes of the tests, three such models were created, different from each other in terms of architecture and the number of hyperparameters and trainable parameters. The described study is a part of the decision-making system, supporting the process of qualitative analysis of the obtained cast iron castings.

## 1. Introduction

The analysis of the image obtained on the basis of an examination carried out with the use of a computer tomograph (CT) allows to estimate the probability of occurrence of defects in the tested castings. The CT is a non-destructive method that allows you to evaluate the product without damaging it (in some cases, because sometimes the sample needs to be cut out, it depends on the thickness of the casting walls and the studied material). The interpretation of the picture is performed by experts. The obtained image is not unambiguous. Its analysis requires a lot of experience, especially in the case of iron castings. A CT tool to take such images and evaluate the information obtained from their manual interpretation was used in this study. The “manual” interpretation of images is performed on a daily basis. In our article we attempted to develop a model using the methods of artificial intelligence, which allows to assess whether the visible area on the photo is a void, crack, or some other detail. It should be noted that the methods of artificial intelligence were used to predict properties as well as evaluate and classify the results of research on metal products. A significant problem when using this type of solution is the amount of data. The use of artificial intelligence or machine learning as well as data analysis to develop predictive models to determine the mechanical properties of products is more and more commonly described in the literature [1,2,3,4,5,6]. For example, a multi-task learning algorithm with augmentation data preprocessing dealt with the small imbalanced data and multi-target predictions. For experiments, negative thermal expansion materials were used. The development of each new model with the use of artificial intelligence to predict material data and its verification using a physical experiment provides a great contribution to the development of the field. Additionally, predicting the mechanical properties of metal products using artificial intelligence methods is a research trend. Classification of the quality of castings based on images (in the indicated cases they were images of microstructure) using artificial intelligence methods is currently an important research trend. Such action is used in negative thermal expansion materials for example on nanoporous metals, with their complex microstructure, ultra-fine-grained FeeC alloy, or for classification of the quality of castings based on images. Review of the literature shows that the use of a tomographic examination and the analysis of tomographic images allows for the classification of the quality of manufactured metal products. Such an analysis allows to indicate the risk of discontinuity that may adversely affect the mechanical properties of the casting. However, no description was found in the literature of an attempt to build such a model in relation to the results of tomographic examinations of iron castings. The development of each new model with the use of artificial intelligence to predict material data and its verification using a physical experiment provides a great contribution to the development of the field. The articles [7,8,9,10,11] present application of analytical electron microscopy and tomographic techniques to perform the qualitative and quantitative characterization of structural elements in the casts which are then analyzed for classification or/and an evaluation of quality products.

## 2. Materials and Methods

### 2.1. Tomograph

The tomographic examination consists of X-rays with X-rays emitted parallel to the image plane of the examined object. As a result of this action, a three-dimensional image is created, which is used to assemble a set of their two-dimensional counterparts (called radiographs), which are recorded at different angles to the adopted coordinate system [12]. Thanks to the high resolution, it is possible to accurately reproduce the complex surfaces of the tested object. This allows for the presentation of internal discontinuities both in two and three dimensions, which allows their exact location and determination of the size [13]. The radiation emitted by the source passes through the object and weakens, and its intensity is converted into grayscale contrast. The weakening of the X-ray beam was characterized using Beer’s law [12].

The volume of the tested object is divided into the so-called voxels, single spatial cells in which the degree of radiation absorption is constant. Each of these voxels can be assigned the diameter of the object divided by the number of pixels. Tomographic images are created in very high resolutions. This is because it is not uncommon for even several thousand two-dimensional photos to be juxtaposed to create a spatial whole. Of course, there are no ideal methods and it is no different in the case of computed tomography. This method is not suitable for testing materials characterized by high density or large wall thicknesses [14,15]. The first of these methods is similar to the system of receptors in the organ of vision, the eyes. The second one, however, has a significant advantage over it, it is simpler and much more convenient, the consequence of which is the fact that it is much more widespread than its hexagonal counterpart. Most of the currently used graphic formats are based on the latter of the described grids. One of the most important parameters of any image is its resolution. It is a compromise measure of the ability to recognize image details [15]. The greater the resolution of an image, the greater the level of detail it represents. Note, however, that a linear increase in resolution results in a square increase in file size. It is also worth mentioning the available color palettes. The measure of their complexity is the number of bits used to remember the state of a single pixel (BPP). The family of algorithms that allow you to perform operations on images is very diverse. It includes, among others, various types of element transformations (scaling, translations, and rotation), pixel brightness transformations (binarization), quality improvement (filtering and artifact removal) and isolation of certain fragments to facilitate the detection of searched objects. In some cases, performing the above-mentioned actions achieves the desired goal, but usually they are part of a larger set of operations and are included in the pre-processing stage.

**Figure 1:**
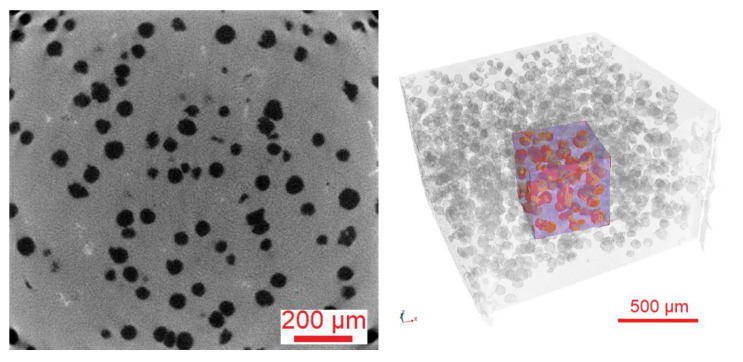
Tomogram showing the microstructure with spheroidal graphite and 3D reconstruction of the sample with the analyzed area.

One of the methods of casting analysis is scanning the examined fragment with X-rays. This method is classified as non-invasive, i.e., the analyzed fragment is not damaged during the test. In practice, it often happens that a selected fragment has previously been specially cut out of the entire casting, which can already be considered an invasive effect on the casting. Nevertheless, this analysis allows for a much more precise dimension of examining the casting fragment, which is not available when examining the photos with a microscope. For this reason, it is an increasingly common form of verification of casting properties. A classic tomograph X-ray examines an object on a 2D scale. A popular solution for capturing radiation is a detector located behind the scanned object. Its task is to measure the intensity of radiation and transmit it as electrical signals. The density of the tested material is of great importance because the higher its value, the less radiation reaches the data-collecting unit, in this case a detector. This means that the material absorbed the radiation that penetrated it. As a result, the material in the photos obtained have a lighter color. Casting defects, e.g., cracks, hardly absorb radiation. For this reason, they are much more visible in X-rays than in places without defects. The cracks in the photos are darker because the increased radiation that they have not absorbed reaches the detector. However, a single X-ray image is not sufficient as it does not provide information on the exact dimensions of the defects (e.g., volume and depth). It is impossible to determine whether the observed defect is located, for example, more to the left or to the right. Additionally, a photo of the casting taken in only one axis may not detect some imperfections. This problem is solved by X-ray computed tomography by taking multiple images of the cast from different angles. The main goal is to create a 3D model of the sample using the photos taken. This approach is most common in medicine. The radiation source and the detector are located in the round part of the device on the movable ring. The lamp together with the detectors takes a photo of the object and then moves a certain distance. In this way, pictures of the object are obtained from different perspectives, which makes it possible to accurately locate possible defects. For the purposes of the article, a tomograph from the Łukasiewicz Research Network-Krakow Institute of Technology, Krakow, Poland (former Foundry Research Institute in Kraków) was used. It consists of an X-ray tube, a detector, a rotating table, and a computer with software enabling the visualization. Tomographs may differ depending on the generation by a different way of realizing the movable lamp-detector system and the arrangement of detectors, but they work in the same way. X-ray computed tomography allows obtaining information about the internal structure of the examined object without interfering with its interior. Unlike a CT scanner used in medicine, in industrial scans the radiation source and detectors are stationary. The test cast is placed on the rotating bottom. First, two-dimensional images of the sample are captured. The radiation generated by the lamp is partially absorbed by the object and then integrated by a detector which converts it into a digital image. The tomograph takes pictures of the sample, and after each of them the element is rotated by a set number of degrees. The three-dimensional image is numerically reproduced from the two-dimensional images. Three-dimensional images, as opposed to two-dimensional ones, provide additional information about the examined object, e.g., they enable the visualization of internal discontinuities of the material. X-ray beam attenuation measurements that fit in each of the voxels are needed to create the image. The measurements are converted into a grayscale contrast that can be seen in 2D images. The direct relation between the local gray level and the degree of light attenuation allows the reconstruction of the mass distribution in the analyzed volume. The obtained image is characterized by very high resolution, which enables precise research and analysis of the structure features of heterogeneous microstructures of materials. The tested object can be displayed in the form of a cross-section, there is also visualization of internal discontinuities, cracks, porosity, and the exact location of the defect. Additionally, it is possible to investigate the distance, volume, and differences in pore density. An example view of the sample and the microstructure of the tested material are shown in Figure 1. 

### 2.2. Tested Material

**Figure 2:**
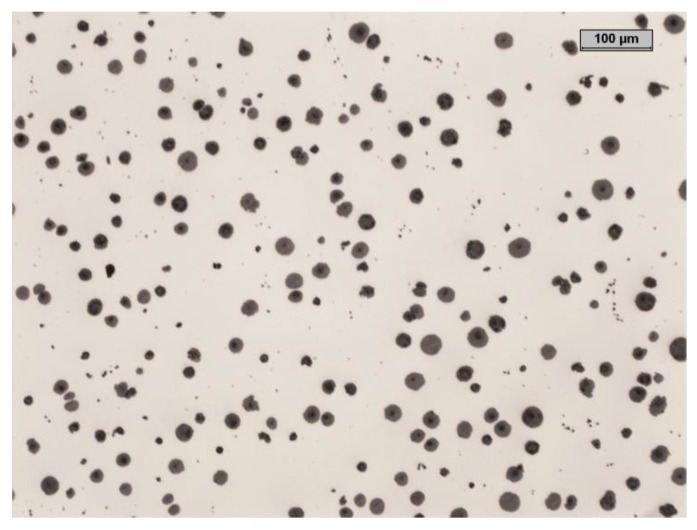
The non-etched microstructure of the investigated cast iron.

**Figure 3:**
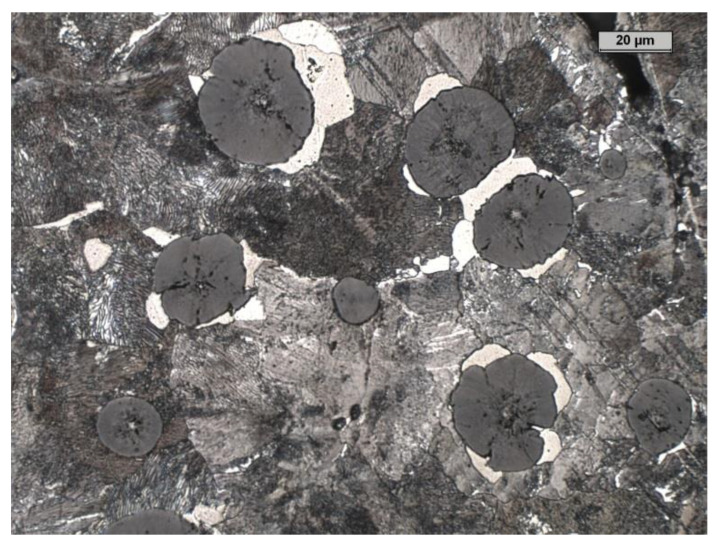
The etched microstructure of the investigated cast iron.

**Figure 4:**
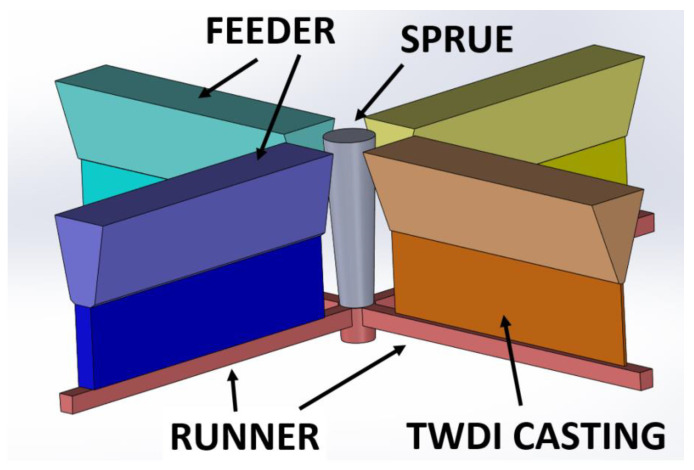
The concept of the 3 layouts of the castings in the mold.

Table 1 and Table 2 show the characteristics of the tested material. Figure 2 and Figure 3 show an example of the microstructure of the tested material. Figure 4 shows the concept of the 3 layouts of the castings in the mold.

Muld design: Castings dimensions: 185 × 50 mm^2^ (+50 mm feeder) 5 mm thickness; plates were placed vertically with the feeder on top, and were cast in green sand-dried molds.

The Table 3 shows chemical composition of the tested material. Table 4 shows CT scanning parameters the tested material. Table 5 shows summary of the results obtained by the CT method for tested material. 

**Figure 5:**
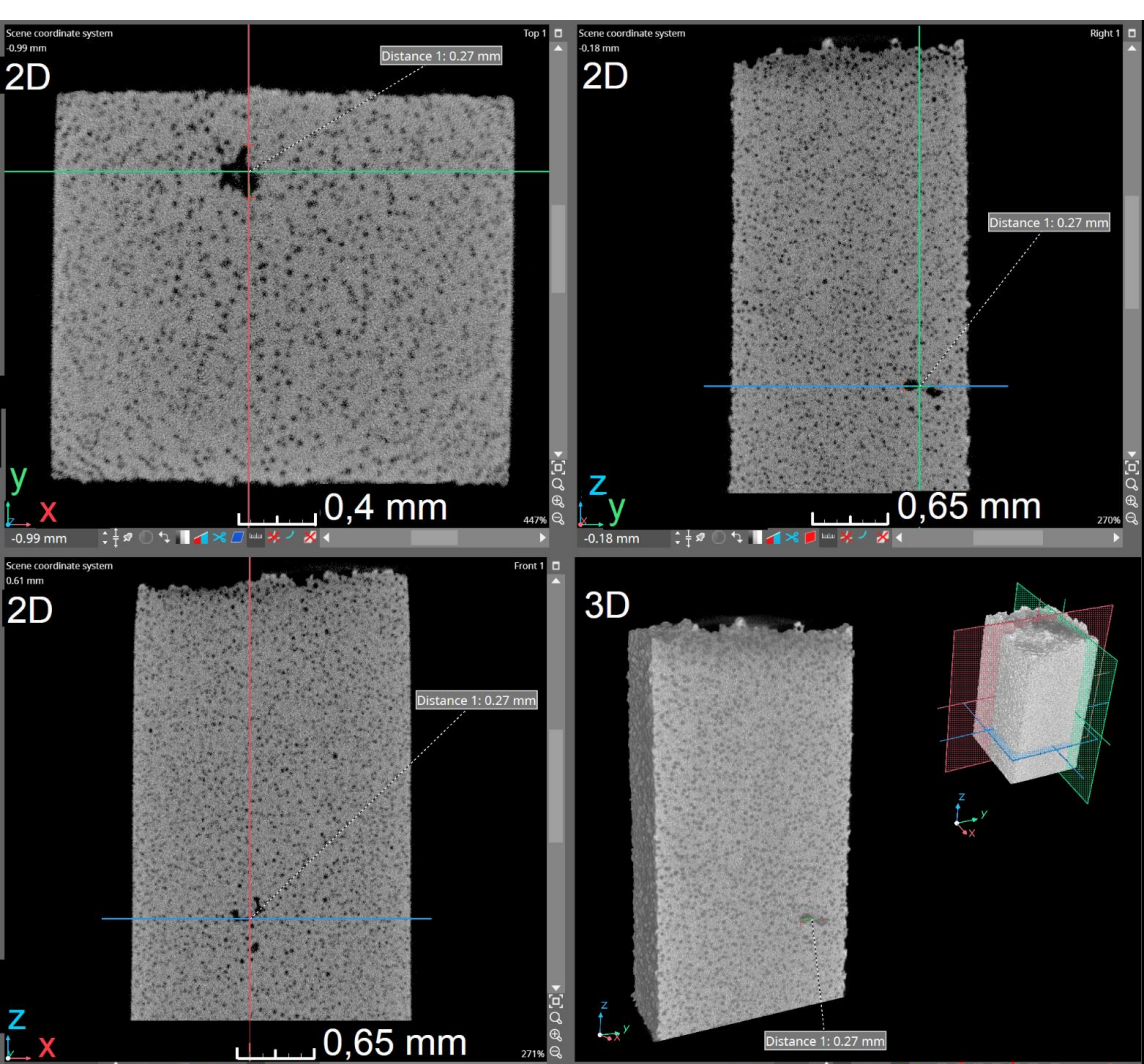
The VGStudio Max software dialog box showing: selected 2D cross-sections (in the xy, yz, and xz plane); 3D spatial section with marked location of section planes. The image shows spheroidal graphite, additionally dimensioning the selected internal discontinuity.

**Figure 6:**
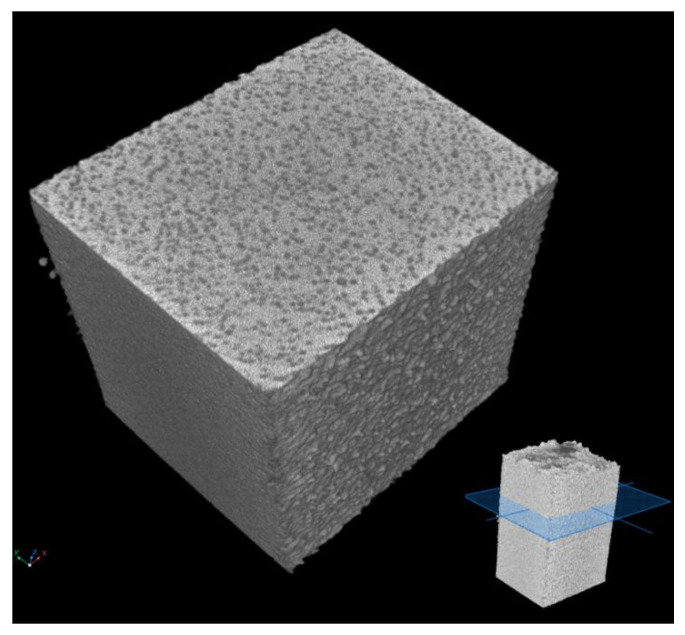
CT image of a ductile iron sample.

**Figure 7:**
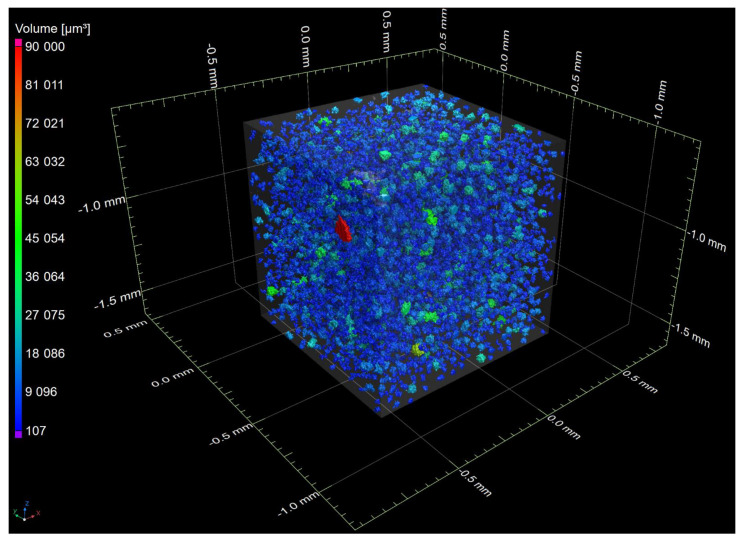
A spatial image showing a fragment of the sample after graphite analysis in ductile iron. Partial transparency of the detail was used.

The tests were performed using a GE X-ray computed tomograph, Nanotom type. The following parameters were used:

The views of the presented solution are shown in the Figure 5, Figure 6 and Figure 7.

### 2.3. Work Scenario

Test scenarios of the module analyzing two-dimensional tomographic images should focus on the comprehensive assessment by convolutional neural network models, which are designed to classify the provided image. For the purposes of the tests, three such models were created, differing from each other in terms of architecture and the number of hyperparameters and trainable parameters.

In order to effectively train the prepared model, it is necessary to have a significant amount of test data, especially when the network is to correctly classify the images in relation to many classes. Too little training data have a negative impact on the learning process. In addition, the training data must not be too similar to each other, as this also results in a significant deterioration of the training quality of the model. Unfortunately, the technique is ineffective when the programmer has a small number of original elements in the collection. The authors of this study did not manage to gain access to the appropriate number of two-dimensional tomographic images that would present the results of tomographic examination of ductile iron castings. It should be borne in mind that this type of data are not common. The authors were able to verify the correctness of the obtained results on only single copy. In order to effectively train the prepared model, it is necessary to have a significant amount of test data, especially when the network is to correctly classify images in relation to many classes. In addition, the training data must not be too similar to each other, as this also results in a significant deterioration of the training quality of the model. It is true that many libraries have implemented the functionality of generating images based on an existing set based on geometric transformations (translations, rotations, etc.). Unfortunately, the technique is ineffective when the programmer has a small number of original elements in the collection. With all this in mind, the authors decided to conduct the training process of the models proposed by him based on data that are in some way similar to those present in the problem under consideration. Originally, the images were in the DICOM format, which is the format characteristic of computed tomography results. In order to facilitate the work with the data from this set, the authors converted them into JPG images (lossless format). The collection contains 1000 unique photos, of which 338 are of defect 1, 187 of defect 2, 260 of defect 3, and 215 of without defect.

### 2.4. Learning Process

Before starting the learning process, the data were pre-processed, which included the following operations:

- Three subsets were separated from the main set: teaching, validation, and testing in the proportion of 70%–10%–20%;
- The image size was standardized to 350 by 350 pixels;
- Converting images to grayscale. Given that CT images are grayscale images, the use of RGB is ineffective. Reducing the dimensions of the color scale representation from three to one significantly improves the efficiency of the training process as the network has less data to process.

Additionally, the so-called confusion matrix was used, which allows you to visualize the quality of predictions made with a specific model.

**Figure 8:**
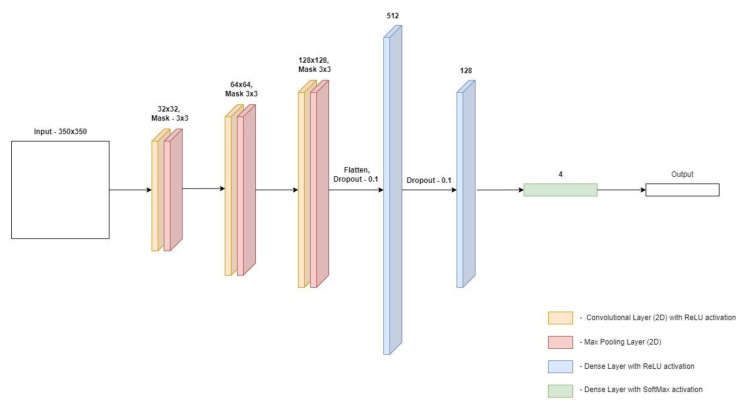
Architecture of the first convolutional neural network model.

The architecture of the first of the proposed models is presented in Figure 8. In first model the input image is 350 × 350 pixels. The model contains three pairs of layers consisting of a convolution layer and a connection layer. A 3 × 3 mask was used in each of the convolution layers, and a standard 2 × 2 mask was used in the joining layers. For both types of layers, the step size was 1. In this model, the number of neurons in the convolution layers was doubled in each dimension, the first layer has a size of 32 × 32, the next one is 64 × 64, and the last one is 128 × 128. The last pair is followed by the flattening of the matrix into a one-dimensional vector, this is performed by the Flatten layer. Then two layers are placed fully connected, containing 512 and 128 neurons, respectively. These numbers are justified because the convolutional layers generate a huge number of neurons which, after flattening, should be connected to the dense layers. The sudden shift in the size of the dense layers allows the problem to be gradually reduced in size. In order to protect the model from overfitting, a dense Dropout layer was applied before each layer, the task of which is to exclude a certain number of neurons from the training process, in this case it applies to 10% of all neurons fed to the Dropout layer. The last element of the model is the dense layer which “selects” one value with the highest score classifying a given photo in the context of specific classes. The Adam algorithm (one of the stochastic simple gradient methods) is responsible for the optimization, and the cost function is the Categorical cross-entropy function.

**Figure 9:**
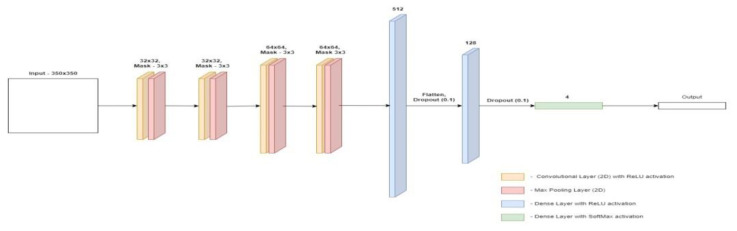
Architecture of the second convolutional neural network model.

The architecture of the second of the proposed models is shown in Figure 9. In the second model, the input image is 350 × 350 pixels. It is processed by four pairs of layers each of them consists of a convolution layer and a bond layer. The sizes of the masks remained unchanged compared with the first model. The pair in which the convolution layer was 128 × 128 was removed from the model. Each of the other convolution layers was duplicated along with the following join layer. The rest of the model remained unchanged compared with its predecessor.

**Figure 10:**
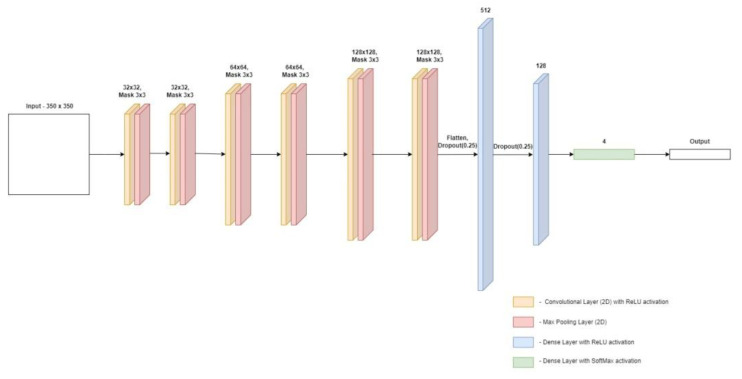
Architecture of the third convolutional neural network model.

The size in last model and the input image is 350 × 350 pixels. Six pairs of layers are responsible for its processing. Compared with the previous model, two new pairs were added, formed by the convolution layer and the fusion layer. The convolution layers of the added pairs have a dimension of 128 × 128 neurons. Due to the increasing complexity of the model, the percentage value in the Dropout layer was increased from 10% to 25%. The rest of the model remained unchanged from its predecessors. The learning process was carried out over 300 epochs. The last model was created using the so-called Hyperparameter Tuning. This process took place in the test range of 50 epochs, and the selection was made of the number of pairs (formed by the convolution layer and the fusion layer) and the number of neurons in a given layer. The determinant for which the selection was made was the measure of accuracy. The architecture of the last of the proposed models is presented in Figure 10. 

The data prepared in this way was processed during the learning process by three models of convolutional neural networks, which were proposed by the authors of this paper. However, it should be noted that the dataset selected by the authors may be considered demanding in terms of classification due to the small amount of data available.

## 3. Results

The evaluation of the models was carried out in a comprehensive manner and concerns both the learning process and subsequent predictions. Two graphs were used to illustrate the learning process, which show the change in accuracy and loss in specific epochs in the context of validation data. The following metrics were used to assess the quality of the model based on the test data: Precision: prediction accuracy within a specific class; recall: the number of recognized items from a specific class; F1: harmonic mean representing the averaging of precision and recall measures. In addition, the so-called confusion matrix was used, which allows you to visualize the quality of predictions made with a specific model.

### 3.1. Results for Model Number 1

**Figure 11:**
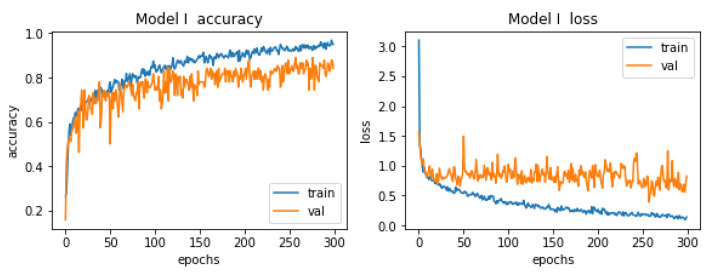
Accuracy and losses when teaching the first model.

In the first model the learning process was carried out on the number of 300 epochs. Graphs showing its course are presented in Figure 11.

The conclusion from these graphs is that there was a slight overfitting during the learning process. The accuracy curves are fine, but the situation is slightly worse for the loss graph. Already in the early epochs, we can observe tendencies that the values of losses on validation data are higher than the values of losses on training data, this is a classic symptom of overfitting. Unfortunately, the reason for the occurrence of this phenomenon lies in the number of paintings that the authors has at his disposal. It contains 1000 items, which is far from the ideal number, there should be several times more of these items. It is hard to choose the so-called the golden age, i.e., the era in which the quality of the model is the highest. There are slight oscillations on both curves (with a few exceptions), which makes choosing the mentioned golden age a difficult task. However, taking into account all the described dependencies, this model cannot be classified as bad, it has a great potential that will be fully released when the training set contains an appropriate number of elements.

**Figure 12:**
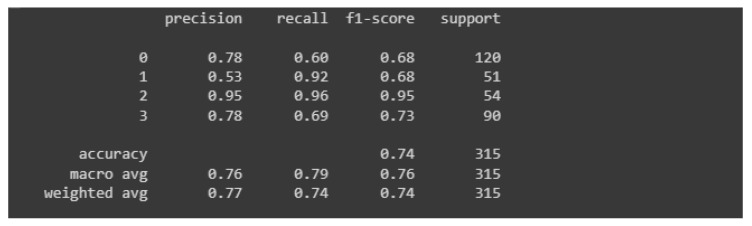
Classification report for the first model.

Figure 12 shows the so-called classification report that shows precision, recall and F1. The “support” column carries information about the number of samples from the test set that participated in the model evaluation.

Despite the occurrence of the overfitting phenomenon, the model has an accuracy of about 74%, which is a positively surprising result (especially considering the occurrence of overfitting). The highest values of measures for classifier evaluation occur in the context of class 2, which means correct castings.

The first model almost flawlessly classifies images of damaged castings and correct castings. The great advantage is that the model almost always classifies damaged castings as damaged castings (there were only three errors, which accounted for 1% of all predictions for damaged parts). The authors assume that it is much better to qualify a good cast as damaged (which is verified anyway) than the other way around. If the defect is defined, the type of defect should also be confirmed, additional measurements should be made and an appropriate description created along with the justification for the decision to disqualify the alloy. 

### 3.2. Results for Model Number 2

**Figure 13:**
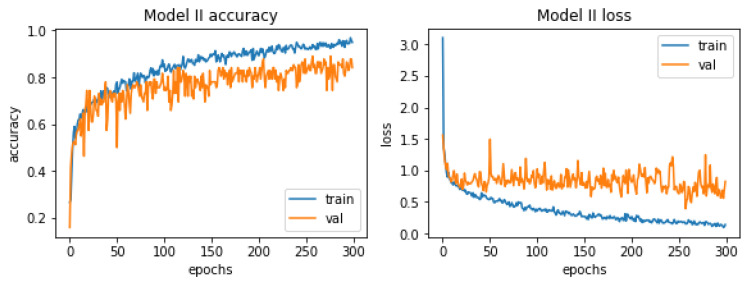
Accuracy and losses when teaching the second model.

Figure 13 shows the charts of accuracy and losses in the context of the learning process. They do not differ much from those relating to the first model. As in the previous case, the phenomenon of overfitting occurs here. The reason for this is the same, too little test data. A slight difference can be seen in the context of the curves representing the accuracy, the one representing the accuracy in the context of the test data is slightly more flattened and over a long period of time it coincides with its validation counterpart.

**Figure 14:**
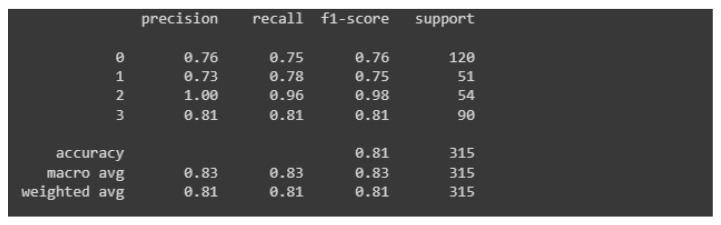
Classification report for the second model.

The learning process was again carried out on the number of 300 epochs. In Figure 14, the classification report is presented, which is a visualization of all measures used to evaluate the classifier.

Based on the classification report, it can be concluded that the latter model is slightly better than its predecessor. The value of each measure is higher than its counterpart from the first model. Precision for images relating to healthy people reached the highest possible value. The accuracy of the entire model is rated at 81%, which is a better result than its predecessor, which had an accuracy of 74%. Again, the model very accurately assesses photos showing castings without defects (two mistakes were made, 4% of all predictions). Importantly, no damaged casting was classified incorrectly.

### 3.3. Results for Model Number 3

**Figure 15:**
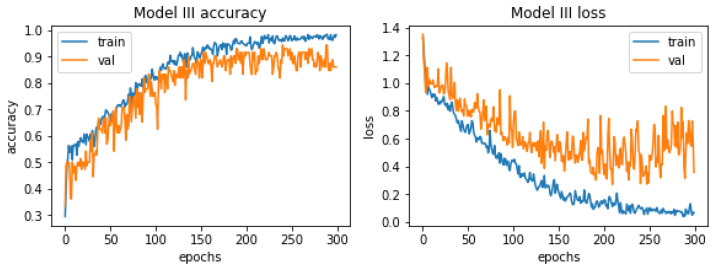
Accuracy and losses when teaching the third model.

The last model quality of this process is visualized in Figure 15.

In this case the model is overfitted almost from the very beginning. It is also highly problematic to identify one golden age. Although the oscillation in the case of the curve representing the losses in the context of validation data may seem larger, in reality it is not true, the scale changed and the validation error is much lower than in the previous models. The highest accuracy value was reached around epoch 290. The harm that represents the accuracy of the learning process with respect to validation values is located very close to its training counterpart, especially in the first half of the learning process.

**Figure 16:**
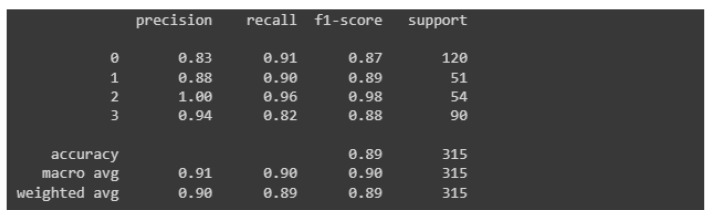
Classification report for the third model.

For the model in question, a classification report was generated, which is presented below in Figure 16.

The values of the measures are by far the highest among all the models presented in this paper. None of these measures has a value lower than 0.8, which is satisfactory in the context of research, the results of which can be applied to industry. Again, the precision measure value hit “1” for an image grade of undamaged castings. The overall accuracy of this model was estimated at 89%, which is much better than its predecessors, which had an accuracy of 74% and 81% respectively.

## 4. Discussion

The characteristics of each model are summarized in tabular form and are presented in Table 6 below.

All the properties and statistics listed in Table 6 come from the classification report and the error matrix. The models were assessed on the set of test data. They were unlabeled and did not take part in the learning process. The first criterion considered was overall accuracy. Each of the obtained results was at least satisfactory in this respect. All models classified the images of the correct quota at the same level of 96% of correct predictions. The most important criterion, images of ductile iron samples with defects, were were found to be free of defects. This mistake can be very fatal. Fortunately, the number of such mistakes was small, they were all made by Model I, where they accounted for 1% of all image predictions. The other models were flawless in this matter.

With all these criteria in mind, it cannot be clearly stated whether Model II is better than Model I and vice versa. Model III improves upon its predecessors in almost every field and is considered by the authors to be the most successful.

## 5. Conclusions

The module described in this paper is a part of the decision support system. The potential user can use it to carry out a comprehensive evaluation of the tested element made of ductile iron, which is the subject of the analysis. This analysis consists of several stages:

- Validation of input data using the so-called “main validators”;
- Validation of the temperatures used during the manufacturing process in the context of the expected alloy grade;
- Assessment of the percentage of the most important elements in relation to the chemical composition of ductile iron;
- Assessment of the length of the austenitization process in relation to the thickness of the sample walls;
- Evaluation of the length of the isothermal transformation process in relation to the wall thickness of the sample;
- Analysis of the image from the computer tomograph for the presence of defects in the sample;
- In this article only the last module is described in detail.

The multifaceted nature of this process guarantees the versatility of the performed analysis process. It is also one of the arguments for the high quality of this study.

The performance tests of the module responsible for the analysis of the sample’s characteristics provided satisfactory results. The entire evaluation process took an average of about 10.5 s, which is a satisfactory result, taking into account the amount of information to be processed by the system and the degree of its complexity. The input data were described along with a brief justification of their choice by the author, and comprehensive tests of the three proposed models, which differed from each other in terms of architecture, were carried out. These tests included the analysis and comparison of several measures, which provided a fair picture of the situation: measures of accuracy, losses, precision, recall, and F1. Additionally, error matrices were made. Based on the tests, it can be concluded that the best model is Model III, which is justified in the study, although each of the proposed network architectures can be considered at least correct. However, taking into account all the results of research and analyses that were carried out using real data and test cases (consulted with employees from the Foundry Research Institute, currently the Łukasiewicz Research Network-Kraków Institute of Technology), the authors of this study consider the operation of both application modules to be satisfactory. The possibility of applying the practical test result requires elaboration. More and more units use a tomography to test the quality of the castings made. The problem is the interpretation of the obtained results; therefore every tool supporting this process is helpful. The use of artificial intelligence in model development is a very dynamically developing field. Each research on real data allows researchers to gain new information and develop this field.

## Figures and Tables

**Table 1:** Charging materials.

Pig iron 4 wt.% C; 0.7 wt.% Si;
Home scrap ductile iron;
Carbourizer;
FeMn80, FeSi75;
99.9 pure Cu and Ni;
Inoculant-Foundrysil (2–7 mm) (FeSi + Ca, Ba)–0.4% mass (Elkem, Oslo, Norway);
Spheroidizing agent-FeSiMg masteralloy Elmag 5800–1.5% mass (Elkem, Oslo, Norway).

**Table 2:** Melting.

Medium frequency induction furnace 50 kg capacity;
Neutral lining;
Overheating temperature—1500 °C;
Tapping temperature (FLOTRET process)—1490 °C;
Pouring temperature—1420 °C.

**Table 3:** Chemical composition.

Chemical Composition, wt.%								
C	Si	Mn	P	S	Mg	Cu	Ni	Cr
3.30	2.60	0.2	0.055	0.006	0.04	0.8	1.5	0.05

**Table 4:** CT scanning parameters..

Voltage = 130 kV;
Current = 35 mA;
Timing = 500 ms;
Voxelsize = 1.25 μm;
NumberImages = 2200.

**Table 5:** Summary of the results obtained by the CT method.

No	Difference Vv [%]	Ductile Iron	Graphite Volume Fraction Vv [%]	
1	1, 3	50%VI4 + 50%V5	11, 3	
2		1, 1	80%VI5 + 20%V6	11, 0

**Table 6:** Summary of model characteristics.

No	Criterion	Model I	Model II	Model III
1	Overall accuracy	74%	81%	98%
2	Good castings considered incorrect	2 z 54% (4%)	2 z 54% (4%)	2 z 54% (4%)
3	Bad castings considered good	3 z 261 (1%)	0 z 261	0 z 261

## Data Availability

Not applicable.
